# Erratum for Zhao et al., “Phenotypic and genotypic differences between bloodstream and non-blood *Candida krusei* isolates: implications for invasiveness and antifungal susceptibility”

**DOI:** 10.1128/spectrum.04111-25

**Published:** 2026-02-13

**Authors:** Ying Zhao, Han Wang, Jinhan Yu, Yi Li, Ge Zhang, Wei Kang, Meng Xiao, Qiwen Yang, Lina Guo, Yingchun Xu

## ERRATUM

Volume 14, no. 1, e02608-25, 2026, https://doi.org/10.1128/spectrum.02608-25. PDF page 14, Funding: “Natural Science Foundation of Henan Province” should read “Natural Science Foundation of China.”

